# Supplementary material for: Light cone cancellation for variational quantum eigensolver in solving noisy Max-Cut
Source: Sci Rep. 2026 Feb 23;16:9597. doi: 10.1038/s41598-025-31798-1 (PMC13009155; doi:10.1038/s41598-025-31798-1)
Supplement: Supplementary file 1 — Supplementary Information. [file 41598_2025_31798_MOESM1_ESM.pdf]

Supplementary Information for Light Cone Cancellation for Variational Quantum Eigensolver in Solving Noisy Max-Cut

Xinwei Lee[1,*,+], Xinjian Yan[2,*,+], Ningyi Xie[2], Yoshiyuki Saito[3], Leo Kurosawa[3], Nobuyoshi Asai[4], Dongsheng Cai[5], and Hoong Chuin LAU[1]

[1]School of Computing and Information Systems, Singapore Management University
[2]Graduate School of Science and Technology, University of Tsukuba
[3]Graduate School of Computer Science and Engineering, University of Aizu
[4]School of Computer Science and Engineering, University of Aizu
[5]Faculty of Engineering, Information and Systems, University of Tsukuba
[*]xwlee@smu.edu.sg
[*]yanxinjian@cavelab.cs.tsukuba.ac.jp
[+]These authors contributed equally to this work.

ABSTRACT

This document contains the supplementary information for the paper "Light Cone Cancellation for Variational Quantum Eigensolver in Solving Noisy Max-Cut".

1 Dataset of the simulations

Table 1 and Table 2 show the simulation datasets used in this study. We consider two categories of unweighted, undirected graphs: the $G(n,p)$ Erdös-Rényi graphs and the regular graphs. The graphs are generated using the `NetworkX` Python package. The $G(n,p)$ graphs are generated using `fast_gnp_random_graph()`; the regular graphs are generated using `random_regular_graph()`, with the seeds specified. Each seed represents one graph instance.

2 Device specifications for the fake backends

This section shows the detailed noise models for the two fake backends used in our demonstrations: `FakeCasablanca` (7 qubits) and `FakeParis` (27 qubits). The basis gates for the two fake devices are the X, $\sqrt{X}$, R_z (rotational Z gate) and the CNOT gate. The R_z gates have zero error. Fig. 1 and 2 show the respective coupling maps for `FakeCascablanca` and `FakeParis`. The CNOT errors for each coupling are shown in Table 3 and 4. Table 5 and 6 show the qubit characteristics for the fake backends `FakeCascablanca` and `FakeParis` respectively. The characteristics listed are the qubit frequencies, T_1 (relaxation time), T_2 (dephasing time), the readout errors, and the single-qubit gate errors (for X and $\sqrt{X}$ gates).

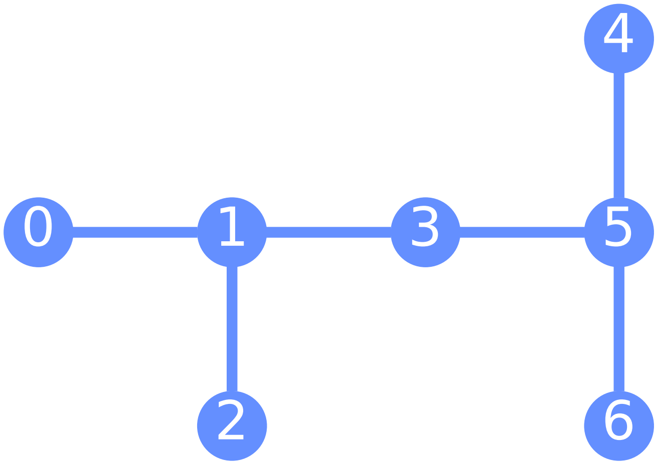

Figure 1. Coupling map of `FakeCasablanca` (7 qubits).

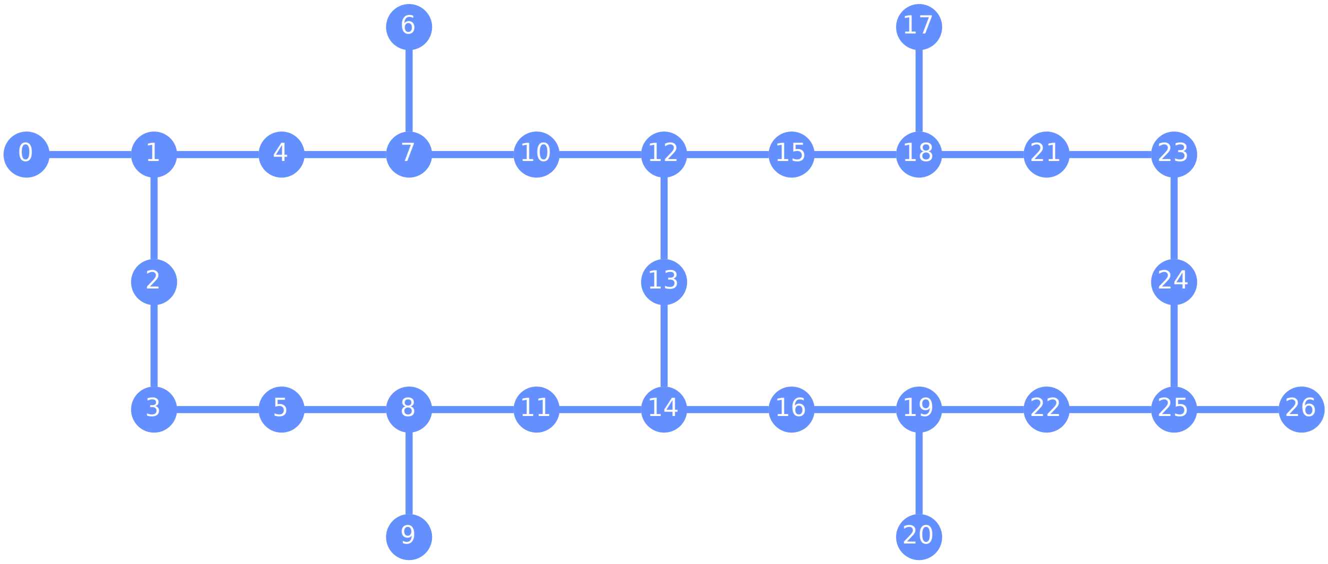

Figure 2. Coupling map of `FakeParis` (27 qubits).

Table 1. The datasets used in the comparative demonstrations of two fake backends—the regular graphs with degree d and $G(n, p)$ graphs with edge probability p.

No. of nodes	Graph type	d (Reg.) or p (ER)	Seed
10	ER	0.5	0, 1, 2, 3
10	Reg.	3	0, 1, 2, 3
11	ER	0.5	0, 1, 2, 3
11	Reg.	4	0, 1, 2, 3
12	ER	0.5	0, 1, 2, 3
12	Reg.	3	0, 1, 2, 3
13	ER	0.5	0, 1
13	Reg.	2	1, 3
14	ER	0.4	0, 1
14	Reg.	2	2, 5
15	ER	0.3	1, 2
15	Reg.	2	3, 7
20	ER	0.25	0, 3, 5, 17
20	Reg.	3	0, 1, 2, 3
30	ER	0.12	0, 5
30	Reg.	2	3, 8
40	ER	0.06	106, 125
40	Reg.	2	0, 1
50	ER	0.06	126, 167, 424, 561
100	ER	0.035	86520, 769454
100	Reg.	3	0, 1

Table 2. The datasets used in the comparative simulations of LCC-VQE and GW algorithm under a noiseless condition.

No. of nodes	Graph type	d (Reg.) or p (ER)	Seed
100	Reg.	3	$0, 1, 2, \ldots, 7$
100	Reg.	4	$0, 1, 2, \ldots, 7$
100	Reg.	5	$0, 1, 2, \ldots, 7$
100	Reg.	6	$0, 1, 2, \ldots, 7$
100	Reg.	7	$0, 1, 2, \ldots, 7$
100	Reg.	8	$0, 1, 2, \ldots, 7$
100	Reg.	9	$0, 1, 2, \ldots, 7$
100	Reg.	10	$0, 1, 2, \ldots, 7$
100	ER	0.1	$0, 1, 2, \ldots, 7$
100	ER	0.2	$0, 1, 2, \ldots, 7$
100	ER	0.3	$0, 1, 2, \ldots, 7$

Table 3. CNOT errors on each qubit coupling for `FakeCasablanca` (7 qubits).

Qubit coupling	CNOT error
(0,1)	1.83×10^{-2}
(1,2)	1.62×10^{-2}
(1,3)	7.49×10^{-3}
(3,5)	1.26×10^{-2}
(4,5)	1.31×10^{-2}
(5,6)	8.70×10^{-3}

Table 4. CNOT errors on each qubit coupling for `FakeParis` (27 qubits).

Qubit coupling	CNOT error
(0,1)	1.26×10^{-2}
(1,2)	1.42×10^{-2}
(1,4)	1.24×10^{-2}
(2,3)	6.52×10^{-2}
(3,5)	1.40×10^{-2}
(4,7)	1.36×10^{-2}
(5,8)	1.29×10^{-2}
(6,7)	1.11×10^{-2}
(7,10)	1.03×10^{-2}
(8,9)	9.56×10^{-3}
(8,11)	7.75×10^{-3}
(10,12)	1.86×10^{-2}
(11,14)	1.20×10^{-2}
(12,13)	1.71×10^{-2}
(12,15)	1.29×10^{-2}
(13,14)	1.18×10^{-2}
(14,16)	1.96×10^{-2}
(15,18)	2.40×10^{-2}
(16,19)	1.32×10^{-2}
(17,18)	2.92×10^{-2}
(18,21)	1.42×10^{-2}
(19,20)	1.32×10^{-2}
(19,22)	1.27×10^{-2}
(21,23)	1.39×10^{-2}
(22,25)	7.60×10^{-3}
(23,24)	1.75×10^{-2}
(24,25)	8.79×10^{-3}
(25,26)	8.01×10^{-3}

Table 5. Qubit characteristics for `FakeCasablanca` (7 qubits).

**Qubit**	**Frequency (GHz)**	$T_1\,(\mu s)$	$T_2\,(\mu s)$	**Readout error**	X **and** $\sqrt{X}$ **gate error**
0	4.82	102.88	53.56	1.97×10^{-2}	3.51×10^{-4}
1	4.76	111.41	109.47	1.44×10^{-2}	8.98×10^{-4}
2	4.91	78.93	132.42	1.78×10^{-2}	3.62×10^{-4}
3	4.88	88.06	77.27	1.56×10^{-2}	2.61×10^{-4}
4	4.87	98.93	39.94	1.54×10^{-2}	5.43×10^{-4}
5	4.96	83.95	122.30	1.74×10^{-2}	4.81×10^{-4}
6	5.18	65.59	63.50	3.26×10^{-2}	4.63×10^{-4}

Table 6. Qubit characteristics for `FakeParis` (27 qubits).

**Qubit**	**Frequency** (GHz)	$T_1\,(\mu s)$	$T_2\,(\mu s)$	**Readout error**	X **and** $\sqrt{X}$ **gate error**
0	5.07	92.03	127.97	2.49×10^{-2}	4.43×10^{-4}
1	5.02	47.59	77.22	1.93×10^{-2}	3.60×10^{-4}
2	4.82	67.94	92.29	1.78×10^{-2}	3.75×10^{-4}
3	4.89	70.03	59.18	1.28×10^{-2}	1.83×10^{-3}
4	5.09	82.70	83.71	2.05×10^{-2}	5.40×10^{-4}
5	4.80	124.04	29.51	2.11×10^{-1}	4.08×10^{-4}
6	5.20	42.87	59.87	1.09×10^{-2}	4.34×10^{-4}
7	5.14	95.38	45.33	2.20×10^{-2}	4.56×10^{-4}
8	5.07	100.04	88.11	1.60×10^{-2}	3.36×10^{-4}
9	5.17	102.78	126.37	1.16×10^{-2}	5.31×10^{-4}
10	4.92	67.52	49.84	1.21×10^{-2}	3.94×10^{-4}
11	4.96	85.88	92.96	2.42×10^{-2}	2.83×10^{-4}
12	5.04	17.98	78.72	2.96×10^{-2}	8.17×10^{-4}
13	5.11	69.75	151.39	1.04×10^{-2}	5.31×10^{-4}
14	4.90	46.68	71.60	1.04×10^{-2}	4.23×10^{-4}
15	4.85	43.04	59.59	1.03×10^{-1}	4.45×10^{-4}
16	5.02	103.24	59.52	4.80×10^{-2}	1.35×10^{-3}
17	5.05	50.87	96.10	3.32×10^{-2}	2.26×10^{-3}
18	4.94	81.45	86.48	1.72×10^{-2}	4.92×10^{-4}
19	4.76	72.08	62.28	9.20×10^{-3}	5.40×10^{-4}
20	5.04	92.45	66.36	1.89×10^{-2}	3.64×10^{-4}
21	4.83	88.85	36.02	5.79×10^{-2}	4.61×10^{-4}
22	4.99	96.86	45.13	1.30×10^{-2}	2.93×10^{-4}
23	5.13	81.18	89.89	1.47×10^{-2}	2.90×10^{-4}
24	4.98	113.32	137.50	1.15×10^{-2}	3.70×10^{-4}
25	4.85	109.80	68.91	3.34×10^{-2}	2.73×10^{-4}
26	4.96	108.41	113.01	6.90×10^{-3}	3.25×10^{-4}
